# Costs of fall injuries in the STRIDE study: an economic evaluation of healthcare system heterogeneity and heterogeneity of treatment effect

**DOI:** 10.1186/s12962-023-00459-3

**Published:** 2023-08-02

**Authors:** David A. Ganz, Thomas M. Gill, David B. Reuben, Shalender Bhasin, Nancy K. Latham, Peter Peduzzi, Erich J. Greene

**Affiliations:** 1 Department of Medicine, David Geffen School of Medicine at UCLA, Los Angeles, CA USA; 2 Geriatric Research, Education and Clinical Center, Veterans Affairs Greater Los Angeles Healthcare System, Los Angeles, CA USA; 3 RAND Corporation, Santa Monica, CA USA; 4 Department of Internal Medicine, Yale School of Medicine, New Haven, CT USA; 5 Boston Claude D. Pepper Older Americans Independence Center, Research Program in Men’s Health: Aging and Metabolism, Brigham and Women’s Hospital, Harvard Medical School, Boston, MA USA; 6 Yale Center for Analytical Sciences, Department of Biostatistics, Yale School of Public Health, New Haven, CT USA

**Keywords:** Fall prevention, Economic evaluation, Heterogeneity of treatment effect

## Abstract

**Objectives:**

The Strategies to Reduce Injuries and Develop Confidence in Elders (STRIDE) Study cluster-randomized 86 primary care practices in 10 healthcare systems to a patient-centered multifactorial fall injury prevention intervention or enhanced usual care, enrolling 5451 participants. We estimated total healthcare costs from participant-reported fall injuries receiving medical attention (FIMA) that were averted by the STRIDE intervention and tested for healthcare-system-level heterogeneity and heterogeneity of treatment effect (HTE).

**Methods:**

Participants were community-dwelling adults age ≥ 70 at increased fall injury risk. We estimated practice-level total costs per person-year of follow-up (PYF), assigning unit costs to FIMA with and without an overnight hospital stay. Using independent variables for treatment arm, healthcare system, and their interaction, we fit a generalized linear model with log link, log follow-up time offset, and Tweedie error distribution.

**Results:**

Unadjusted total costs per PYF were $2,034 (intervention) and $2,289 (control). The adjusted (intervention minus control) cost difference per PYF was -$167 (95% confidence interval (CI), -$491, $216). Cost heterogeneity by healthcare system was present (p = 0.035), as well as HTE (p = 0.090). Adjusted total costs per PYF in control practices varied from $1,529 to $3,684 for individual healthcare systems; one system with mean intervention minus control costs of -$2092 (95% CI, -$3,686 to -$944) per PYF accounted for HTE, but not healthcare system cost heterogeneity.

**Conclusions:**

We observed substantial heterogeneity of healthcare system costs in the STRIDE study, with small reductions in healthcare costs for FIMA in the STRIDE intervention accounted for by a single healthcare system.

**Trial registration:**

Clinicaltrials.gov (NCT02475850).

**Supplementary Information:**

The online version contains supplementary material available at 10.1186/s12962-023-00459-3.

## Introduction

Recommendations for economic evaluation of healthcare interventions emphasize the importance of assessing for heterogeneity of economic outcomes across jurisdictions [1], with the research literature providing both methodology for assessing heterogeneity and empirical examples [2–4]. Although the term “jurisdiction” has been defined broadly, referring to any entity (local or national) needing location-specific economic outcomes, studies tend to place emphasis on between-country comparisons [5], with less attention having been devoted to within-country variation [6]. Within the United States (US), prior work has shown significant regional variation in the cost of usual care [7, 8]. Such work highlights the potential for economic outcomes to vary in multicenter clinical trials conducted across different parts of the United States.

Injuries related to falls are common in the older adult population, leading to an estimated 2.8 million emergency department visits and 800,000 hospital stays in the US annually, and to an annual cost of $49.5 billion [9, 10]. The Strategies to Reduce Injuries and Develop Confidence in Elders (STRIDE) study tested whether a patient-centered multifactorial intervention, delivered by nurses working in primary care settings and trained as falls care managers, would reduce serious fall injuries in adults age 70 and older who were at increased risk for injurious falls. STRIDE cluster-randomized 86 primary care practices in 10 US healthcare systems to either the STRIDE intervention or enhanced usual care, enrolling 5,451 participants. The hazard ratio for first adjudicated serious fall injury (primary outcome) for the intervention compared to enhanced usual care was 0.92 (95% confidence interval [CI], 0.80 to 1.06) [11]. The effect size for other related outcomes, including rates of falls, fall injuries, and fractures, was generally congruent with the primary outcome [11, 12]. Rates of all-cause hospitalization or death were similar between the intervention and enhanced usual care groups [11].

Although STRIDE’s results are compatible with no intervention effect, or even a small increase in serious fall injuries in the intervention group, our best estimate of the intervention’s effect is a reduction in serious fall injuries of 8%, a difference that the study was not statistically powered to detect. If these lower rates of fall injuries observed in STRIDE’s intervention group are real, they could potentially produce meaningful savings to healthcare payers at the population level. To better understand the potential impact on payer cost if the STRIDE intervention were disseminated, we undertook an analysis to estimate potential healthcare costs averted by the STRIDE intervention. We hypothesized that healthcare costs would be lower in the intervention group compared to enhanced usual care, of a similar magnitude to the 8% reduction observed in the primary outcome, although we did not expect this result to be statistically significant. Given known regional variation in United States healthcare utilization patterns [7], we also explored healthcare-system-level heterogeneity in healthcare costs and heterogeneity of treatment effect (HTE), which, if present, might affect decision-makers’ willingness to implement the intervention in different locations. We hypothesized that there would not be statistically significant healthcare-system-level heterogeneity in healthcare costs or HTE. To gain insight about sources of heterogeneity where present, we also evaluated potential mediators of heterogeneity.

## Methods

### Overview

STRIDE’s design, screening and recruitment procedures, intervention, strategies for participant retention, protocol for outcome adjudication, and clinical outcomes have been described previously [11–18]. STRIDE’s 10 healthcare systems (Essentia Health; HealthCare Partners; Johns Hopkins Medicine; Mercy Health; Michigan Medicine, Mount Sinai Health; Partners Healthcare; Reliant Medical Group; University of Pittsburgh Healthcare; and University of Texas Medical Branch Galveston) were geographically diverse, including urban, suburban and rural locations, and had varying payer mix [13]. On March 11, 2015, primary care practices were cluster randomized in a 1:1 ratio to either the STRIDE intervention or enhanced usual care (hereafter referred to as the control group) using covariate-based constrained randomization stratified by healthcare system [11, 13]. Individuals were eligible for participation in STRIDE if they answered “yes” to at least one of three items: (a) have you fallen and hurt yourself in past year?, (b) have you fallen ≥ 2 times in past year?, and (c) are you afraid that you might fall because of balance or walking problems? The STRIDE multifactorial intervention included protocols for assessing and managing strength, gait and balance; medications; osteoporosis and vitamin D; feet and footwear; home safety; postural hypotension; and visual impairment [15]. Participant enrollment ended on March 31, 2017, and the last day of follow-up was March 31, 2019 [14, 16]. STRIDE was approved by a single institutional review board at the Massachusetts General Brigham Healthcare System, Boston, MA, and STRIDE’s statistical analysis plan is available at clinicaltrials.gov [19]. The STRIDE dataset is available in the National Institute on Aging repository [20]. The healthcare utilization and cost analyses described here were not pre-specified but built on the pre-existing statistical analysis plan wherever possible (e.g., in selection of covariates for models). The current work follows Consolidated Health Economic Evaluation Reporting Standards (CHEERS) 2022 guidance (see Supplementary Appendix) [21].

### Data sources

#### Participant interviews

Self-report data from telephone interviews of participants (or proxies) every four months served as the primary source of information on fall-related outcomes. Participants were mailed calendars on which to prospectively record their falls, with these calendars serving as a memory aid during the interview. Trained interviewers from the Yale Recruitment and Assessment Center, who were masked to treatment assignment, carried out the interviews. During these interviews, participants were asked, “Have you fallen in the past four months (or since last contact)?” and if yes, were asked, “How many times have you fallen?” Participants who noted that they had fallen were then asked if they were injured in any fall in the past four months (or since last contact), and if yes, how many falls led to an injury. For every fall that led to an injury, participants were then asked about their use of health care: “Did you see a doctor or other health care professional for the injury?” We used this question to identify fall injuries requiring medical attention (FIMA). Participants were additionally asked, “Were you admitted for an overnight stay, or longer, in the hospital following your injury?” This question allowed us to classify FIMA into events with or without hospitalization.

During interviews, participants were also asked details about the type of injuries incurred for each fall injury event. For descriptive purposes, we categorized events based on the most definitive injury reported, using the following hierarchy: (1) hip fracture, (2) other fracture, (3) dislocation, (4) cut with evidence of closure, or (5) head injury; sprain or strain; bruising or swelling; cut without closure; or other injury.

#### Adjudicated data

FIMA potentially meeting STRIDE’s primary outcome definition of a serious fall injury were further investigated by obtaining at least one additional confirmatory source of data (administrative claims and/or encounter data, or full text of medical records) [17]. Adjudicated serious fall injuries were defined as falls resulting in: [1] billable medical attention according to US Medicare guidelines with (a) fracture (excluding isolated thoracic vertebral and/or lumbar vertebral fracture), (b) joint dislocation, or (c) cut requiring closure; OR [2] overnight hospitalization with (a) head injury, (b) sprain or strain, (c) bruising or swelling, or (d) other injury determined to be “serious” (i.e., burn, rhabdomyolysis, or internal injury) [17]. Adjudication data included information about whether there was an overnight hospitalization for the injury event, and information to classify the most definitive injury for the event using the same five-level hierarchy described for FIMA above.

### Outcomes

FIMA, based on self-report alone, served as the primary outcome for measurement of healthcare utilization and costs. FIMA represent the broadest possible measure of health care utilization available in STRIDE, representing all fall injury events leading to receipt of healthcare. Due to resource constraints, only FIMA that might meet the STRIDE definition of serious fall injury (as defined above) were adjudicated. In supplementary analyses, we evaluated the subset of FIMA that were adjudicated and confirmed to be serious. Although an objective data source, this subset represents a less comprehensive measure of utilization (not representative of all costs).

### Perspective of evaluation

This study takes the perspective of the healthcare payer, representing a decision-maker that could potentially support reimbursement for a program modeled on STRIDE (the primary cost of the program—nurse care manager time to deliver the intervention—is currently non-reimbursable by medical insurance in the US). It is possible that some downstream costs that are reimbursable by healthcare payers resulted from the STRIDE intervention, such as increased use of physical therapy or eye care, but STRIDE did not collect data on these items. Given the marked heterogeneity in costs of care for FIMA observed in the current study (discussed later), we chose to focus this analysis on healthcare use for fall injuries, and the implications of such findings for payers and healthcare systems. Program costs were not included in the evaluation.

### Calculation of costs

We used cost data from Bohl et al. to estimate quarterly costs for hospitalized and non-hospitalized people with falls resulting in medical attention, using the modeled component of costs attributable to the index fall [22]. We inflated Bohl’s cost estimate to 2017 US dollars (the midpoint of the STRIDE study) using the medical care component of the US Consumer Price Index [23]. Quarter 1–4 costs (representing sequential three-month periods since the date of the index fall) in 2017 US dollars were $2,084, $855, $521, and $1,003 for non-hospitalized fallers, and $36,338, $4,402, $2,689, and $2,600 for hospitalized fallers, respectively. We selected Bohl et al. as our source for costs because these data were provided at quarterly rather than annual resolution, allowing more precise modeling of costs over time. The values taken from Bohl et al. are of similar magnitude to other studies that provide annual cost data, although exact results differ due to different datasets and analytic methods [24, 25]. In a sensitivity analysis, we used less detailed but nationally representative costs (available for the one-year period since the date of the index fall) from Medicare data to assess the robustness of our primary findings [24].

We attributed quarterly costs to fall injury events in STRIDE starting at the index date of the fall injury. Consistent with Bohl et al., we allowed costs to extend 12 months from the index date [22], except if participants died, were lost to follow-up, or had a recurrent fall injury event, in which case costs were prorated to reflect time until the relevant date. If a STRIDE participant had a recurrent fall injury event, fall costs were “reset” with the new index date, and a fresh set of costs was incurred. The time horizon used was that of the clinical trial itself, where participants were followed for a median of 2.3 years (interquartile range, 2.0-2.7 years) [12]. Given the short time horizon, we did not apply a discount rate to the results.

### Statistical analysis

The primary unit of analysis (and inference) in this study was the primary care practice. Primary care practices were the units of randomization in STRIDE and represent a level at which healthcare payers might assess utilization and cost information as a measure of a practice’s efficiency [26]. Of note, STRIDE did not have access to provider-level information. We computed descriptive statistics about the practices and their participants using counts, median/interquartile range, and mean/standard deviation, as appropriate.

We calculated unadjusted total costs of fall injuries per person-year of follow-up (PYF), with follow-up time defined as the time from a participant’s enrollment in the study to their last follow-up interview. Normalizing by PYF was necessary because practices varied in the number of enrolled participants per practice, and participants varied in their duration of follow-up time, due to being enrolled in the study at different times. To calculate total costs per PYF, we assigned a cost to each unit of utilization (fall injury event) as noted above and summed all such costs at the level of the participant. We then summed all participant-level costs within each practice to obtain the total cost per practice. We calculated the total PYF for each practice as the sum of PYF for all study participants assigned to each practice.

We conducted our primary adjusted analyses using a generalized linear model with a Tweedie error distribution, log link, and natural log of PYF as offset. Tweedie models are useful for non-negative outcome data with a potential mass at zero and rightward skew, characteristic of cost data [27]. We inspected a normal probability plot of residuals to verify adequate model fit. In a sensitivity analysis, we fit a model with a negative binomial error distribution, log link, and log PYF offset.

All models adjusted for study design, including fixed effects for treatment arm, healthcare system, and interaction of healthcare system with treatment arm. Models also included fixed effects for the constrained randomization variables: (a) practice size (by tertile), (b) geography (urban versus rural), and (c) practice race/ethnicity (majority of patients’ primary identification: nonwhite versus white). The modeled outcome of interest was the intervention minus control group difference in total costs per PYF. To calculate this difference, we used predictive margins with observed covariate patterns. We used bootstrap procedures to estimate 95% confidence limits around the difference.

With respect to statistical inference, we focused on three sets of variables: (a) treatment arm, (b) healthcare system (to determine if heterogeneity of costs by healthcare system existed), and (c) the interaction of healthcare system with treatment arm (to assess for HTE). Since healthcare system was a categorical variable, individual healthcare systems and their interactions with treatment arm were dummy-coded in the model, and the overall effect of healthcare system and its interaction with treatment arm were assessed by omnibus Wald tests across their respective sets of dummy variables.

Because we detected heterogeneity in cost of FIMA by healthcare system and HTE, we conducted additional analyses specific to each healthcare system. Using the fully interacting models noted above, we used predictive margins to generate predicted costs for each healthcare system, by treatment arm. We then calculated the intervention minus control differences in cost, with 95% confidence intervals, using predictive margins and bootstrapping. Noting that a single healthcare system had markedly different results than the others, we carried out a sensitivity analysis to observe which effects would remain if this healthcare system were removed from the analysis.

We also conducted an analysis to assess for potential mediators of healthcare system heterogeneity. We postulated that heterogeneity could be mediated by three primary factors that would act as drivers of costs. First, since fall injury rates are positively associated with fall rates [28], we calculated the total number of falls per PYF for each practice, hypothesizing that there might be residual imbalances in falls per PYF by healthcare system not accounted for in STRIDE’s screening and recruitment process, which selected (at the participant level) for individuals at higher risk of fall injuries [14]. Such healthcare system imbalances could be due to regional variation in environmental factors (like weather conditions) that might influence fall risk [29] or residual participant-level differences in fall risk by healthcare system. Second, we postulated that care-seeking behavior, defined as the ratio of the count of FIMA divided by the count of falls at the practice level, might influence costs. That is, for any given fall event, the propensity to seek medical attention might vary by healthcare system, as care-seeking behavior in general has been shown to vary regionally in prior work [7]. Such differences in seeking medical attention could be due to underlying differences in participant injury rates subsequent to a fall or differences in the rates with which participants seek care for milder injuries. Third, we postulated that treatment intensity, defined as the ratio of FIMA leading to an overnight hospital stay divided by all FIMA, might influence costs and vary at the healthcare system level. The decision to hospitalize has also been shown to vary regionally in prior work and tends to be driven more by supply-side (i.e., provider) factors than care-seeking [7].

For the mediation analysis, we used the R medflex package [30] to model the natural direct (unmediated) and indirect (mediated) effects of healthcare system on cost of FIMA per PYF. These tests were again omnibus Wald tests across the dummy variables for the direct and indirect effects in each healthcare system. With only 86 units of observation (primary care practices), we focused on mediation of healthcare system cost heterogeneity and did not include a treatment arm by healthcare system interaction to avoid over-fitting the mediation models.

For omnibus tests of main effects, p < 0.05 was considered significant; for HTE, we used p < 0.10 [31, 32]. Given the exploratory nature of analyses, we did not adjust for multiple comparisons; results are presented as point estimates with 95% CIs. All models were run in SAS/STAT version 15.2, with the exception of mediation analyses, which were run in R 4.2.1 using version 0.6-7 of the medflex mediation package [30].

## Results

Table 1 shows descriptive characteristics of the 86 practices in STRIDE. Practices appeared balanced on key characteristics at baseline, including those of enrolled participants. Table 2 and Supplementary Table 1 provide counts (and incidence rates) of FIMA and adjudicated serious fall injuries in intervention and control practices during follow-up, respectively, by injury type and whether the participant reported being hospitalized. Differences were small but generally favored the intervention practices.

**Table 1:** Characteristics of the Randomized Practices and the Practice-Level Baseline Characteristics of the Participants Enrolled in the Intervention and Control Practices*

	*Intervention practices (N = 43)*	*Control practices (N = 43)*
**RANDOMIZED PRACTICES**		
Urban, n	39	39
Majority white, n	35	35
Majority English-speaking^†^, n	40	40
Practice size^‡^		
First tertile (400–690)^‡^, n	14	14
Second tertile (694–965)^‡^, n	14	15
Third tertile (985–5946)^‡^, n	15	14
Median (IQR)	772 (524)	802 (611)
**PARTICIPANTS AT BASELINE** ^**¶**^		
Age (years), mean	79.7 ± 1.4	79.3 ± 1.2
Female sex (mean %)	64.2 ± 9.3	63.0 ± 10.9
Race, (mean %)		
White	89.1 ± 14.4	89.2 ± 11.2
Black	6.1 ± 12.1	6.6 ± 9.5
Other	4.3 ± 6.0	3.4 ± 5.0
Unknown	0.4 ± 0.7	0.8 ± 1.1
Latino/Hispanic ethnicity (mean %)	9.7 ± 17.7	9.6 ± 17.9
Education (mean %)		
High school graduate or less	25.5 ± 14.2	26.3 ± 16.0
Some college or equivalent	26.2 ± 9.8	25.5 ± 9.2
College graduate	18.7 ± 7.6	18.6 ± 6.1
Post-graduate	29.6 ± 14.7	29.5 ± 18.0
Unknown	0.0 ± 0.2	0.1 ± 0.4
Chronic conditions^§^, mean	2.1 ± 0.2	2.2 ± 0.2
Cognitively impaired^II^ (mean %)	2.8 ± 2.4	2.8 ± 2.4
Use of mobility aid or nonambulatory (mean %)	34.6 ± 9.2	34.9 ± 9.1
Screening questions for fall injuries (mean %)		
Fell 2 or more times in past year	36.3 ± 8.7	34.8 ± 9.0
Fell and hurt self in past year	38.4 ± 5.2	39.4 ± 6.2
Afraid of falling because of walking or balance problems	86.1 ± 5.4	86.6 ± 6.9

Legend:

* Because the unit of randomization was the practice rather than the participant, this table is included as a check on the adequacy of the randomization

^†^ Not explicitly constrained, balance forced by constraining on rural/urban and majority white

^‡^ The practice size refers to the number of age-eligible patients in the practice. The range of the number of age-eligible patients in each tertile is shown in the parentheses

^¶^ Data for baseline characteristics are mean ± SD

^§^ Chronic conditions included hypertension, fracture other than hip since age 50, cancer, arthritis, diabetes, chronic lung disease, myocardial infarction, stroke, congestive heart failure, hip fracture, and Parkinson’s disease

^II^ Four or more errors on 6-item Callahan cognitive screener or interview completed entirely by proxy

**Table 2:** Counts and incidence rates of FIMA, hierarchically organized by most definitive injury type for each event.*

Injury type	Hospitalized Count (Incidence Rate)**		Not hospitalized Count (Incidence Rate)**	
	Intervention	Control	Intervention	Control
Hip fracture	40 (0.63)	54 (0.89)	3 (0.05)	4 (0.07)
Other fracture	104 (1.64)	91 (1.51)	176 (2.78)	181 (3.00)
Dislocation	2 (0.03)	1 (0.02)	13 (0.21)	13 (0.22)
Cut with evidence of closure	12 (0.19)	21 (0.35)	104 (1.64)	91 (1.51)
All other injuries	129 (2.04)	137 (2.27)	496 (7.83)	484 (8.01)

Abbreviations: FIMA, fall injuries with medical attention

*All injury events are placed into the most definitive category for which they are eligible, ordered from most to least definitive: (1) hip fracture, (2) other fracture, (3) dislocation, (4) cut with evidence of closure, or (5) all other injuries

**Incidence rate is per 100 person-years of follow-up (PYF). The intervention group had a total of 6338.31 PYF; the control group had a total of 6042.51 PYF

**Fig. 1:**
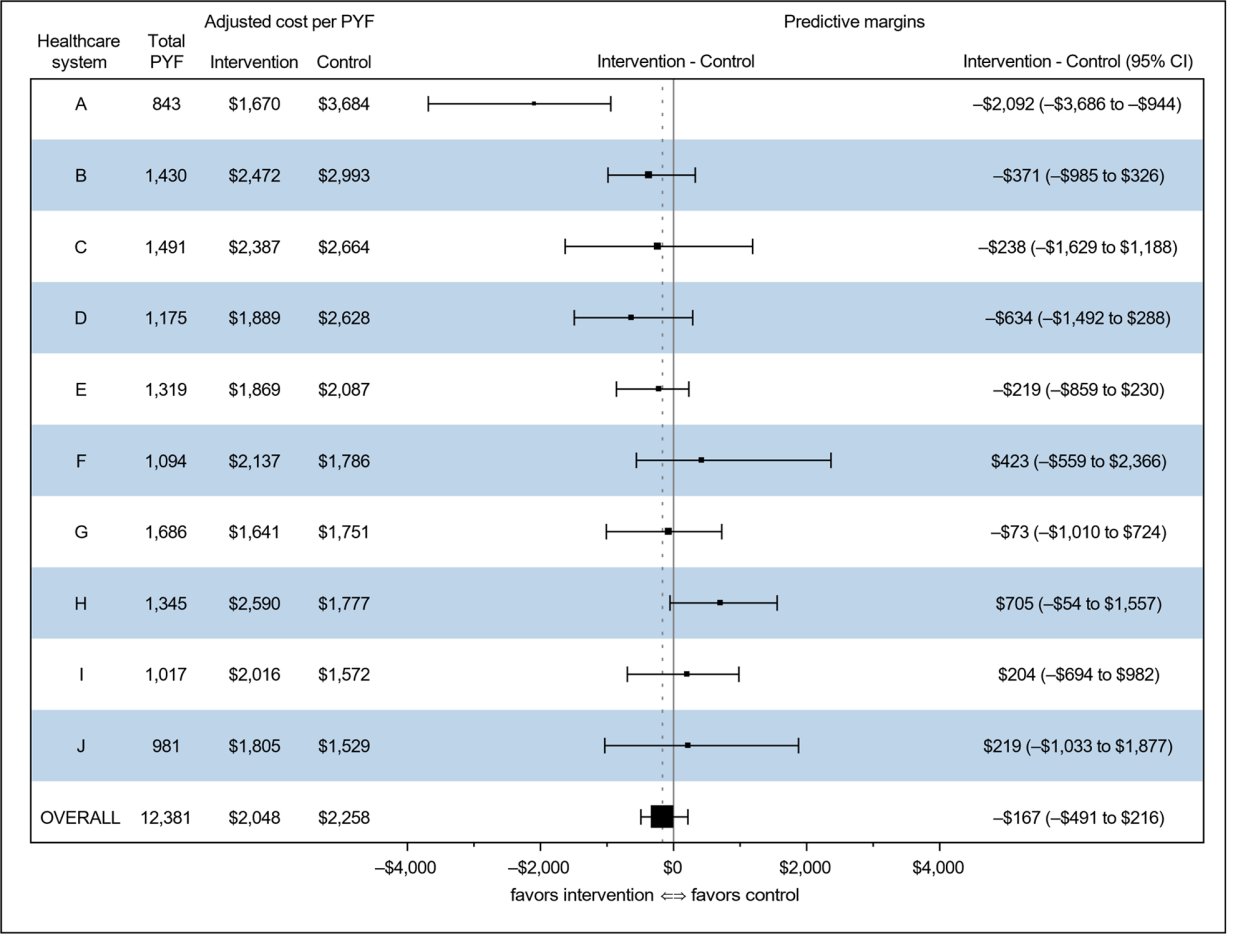
Forest plot showing adjusted intervention minus control costs by healthcare system Healthcare systems are ranked from top to bottom on unadjusted costs of control practices. “Total PYF” represents the total person-years of follow-up for each healthcare system. Adjusted intervention and control costs represent values generated from the Tweedie model. Forest plot and accompanying predictive margin results represent model-generated intervention minus control costs based on actual and counterfactual treatment assignments, with the 95% confidence interval derived from bootstrapping

Supplementary Table 2 shows unadjusted total costs per PYF in intervention and control practices for FIMA and adjudicated serious fall injuries. Table 3 and Supplementary Table 3 show adjusted costs for FIMA and adjudicated serious fall injuries, respectively. Overall unadjusted total costs per PYF for FIMA were $2,034 (intervention) and $2,289 (control); adjusted costs were similar. However, individual healthcare systems demonstrated marked variation in costs per PYF, both in intervention and control practices. These differences persisted after adjustment for variables used in constrained randomization and for healthcare system and healthcare system by treatment arm interaction, with adjusted total costs per PYF in control practices ranging from $1,529 (95% CI, $949 to $2,454) to $3,684 (95% CI, $2,936 to $4,254) for individual healthcare systems. Table 4 shows cost heterogeneity by healthcare system (p = 0.035), as well as HTE (p = 0.090), a finding confirmed in negative binomial models run as a sensitivity analysis. Supplementary Table 4 shows that cost heterogeneity by healthcare system persisted using an alternative data source for costs (p = 0.037), but no HTE was detected (p = 0.258). Figure 1 graphically depicts treatment effects by healthcare system, demonstrating both qualitatively and quantitatively different findings across healthcare systems; however, only healthcare system A shows a confidence interval that does not span zero (intervention minus control costs, –$2,092; 95% CI, –$3,686 to –$944). A sensitivity analysis removing healthcare system A reduced HTE (p = 0.683), but healthcare system cost heterogeneity persisted (p = 0.043). In addition, costs were no longer definitively lower in the intervention practices than in controls, with the Tweedie model showing lower costs for the intervention practices and negative binomial model showing lower costs for controls (results not shown).

**Table 3:** Adjusted total costs per person-year of follow-up for fall injuries leading to medical attention

	Total PYF	Adjusted total costs/PYF (95% CI)**	
Healthcare system*		Intervention	Control
A	843	$1,670 ($1,080-$1,970)	$3,684 ($2,936-$4,254)
B	1,430	$2,472 ($2,215-$2,714)	$2,993 ($2,414-$3,499)
C	1,491	$2,387 ($1,865-$2,938)	$2,664 ($1,493-$4,053)
D	1,175	$1,889 ($1,184-$2,802)	$2,628 ($1,923-$3,293)
E	1,319	$1,869 ($1,266-$2,270)	$2,087 ($1,969-$2,209)
F	1,094	$2,137 ($1,597-$3,862)	$1,786 ($877-$2,667)
G	1,686	$1,641 ($789-$2,108)	$1,751 ($1,050-$2,273)
H	1,345	$2,590 ($2,123-$3,052)	$1,777 ($1,334-$2,110)
I	1,017	$2,016 ($1,232-$2,800)	$1,572 ($1,291-$1,774)
J	981	$1,805 ($917-$2,751)	$1,529 ($949-$2,454)
***Overall***	***12,381***	***$2,048 ($1,856-$2,238)***	***$2,258 ($2,011-$2,558)***

*Healthcare system letters are labeled “A” through “J” based on unadjusted total costs of their control practices, ordered from highest to lowest

**95% CI based on bootstrapped estimates from Tweedie model including covariates used in constrained randomization, treatment arm, and dummy indicators for healthcare systems and their interactions with treatment arm

PYF, person-year of follow-up; CI, confidence interval

**Table 4:** Omnibus (Type 3) Wald tests for cost of FIMA, in Tweedie and negative binomial models

All healthcare systems	Tweedie	negative binomial
*Effect*	*p value*	*p value*
Treatment arm (intervention vs. control)	0.340	0.982
Healthcare system (dummy-coded)	0.035	0.002
Healthcare system by treatment arm interaction	0.090	0.065
Tertile of practice size (dummy-coded)	0.043	0.053
Study participants in practice were majority white race (vs. not)	0.429	0.318
Urban practice (vs. rural)	0.337	0.698
**Sensitivity analysis: healthcare system A excluded**	**Tweedie**	**negative binomial**
*Effect*	*p value*	*p value*
Treatment arm (intervention vs. control)	0.954	0.453
Healthcare system (dummy-coded)	0.043	0.004
Healthcare system by treatment arm interaction	0.683	0.343
Tertile of practice size (dummy-coded)	0.014	0.022
Study participants in practice were majority white race (vs. not)	0.421	0.342
Urban practice (vs. rural)	0.115	0.309

This table shows results for the main analysis that includes all 10 healthcare systems, and a sensitivity analysis where healthcare system A (which showed a confidence interval that excluded zero in favor of the intervention) is excluded

Abbreviations: FIMA, fall injuries with medical attention

Table 5 shows results of omnibus Wald tests from mediation analyses. There was no evidence for a mediation effect of healthcare system heterogeneity by fall risk (p = 0.283), care-seeking behavior (p = 0.431), or treatment intensity (p = 0.057).

**Table 5:** Results of omnibus Wald tests from Tweedie models testing for mediation*

Covariates	Mediator		
	Model 1: Fall risk p value	Model 2: Treatment-seeking p value	Model 3: Treatment intensity p value
Treatment arm (intervention vs. control)	0.337	0.366	0.409
Healthcare system – direct effect	0.010	0.005	0.237
Healthcare system – indirect (mediated) effect	0.283	0.431	0.057

*In addition to covariates shown, models control for constrained randomization variables: (a) practice size (by tertile), (b) geography (urban versus rural), and (c) practice race/ethnicity (majority of patients’ primary identification: nonwhite versus white)

## Discussion

In this economic evaluation, we found that the STRIDE intervention led to small reductions in overall estimated costs relative to controls. However, this average result was driven by a single healthcare system (system A). Healthcare system A was the smallest (in PYF), and its control practices were the highest in adjusted costs, while its intervention practices were second-to-lowest in adjusted costs. From our data it is not possible to determine to what degree results in healthcare system A were due to unusually high-cost control practices, an unusually large intervention effect, or a combination of both. Future work should evaluate healthcare system A’s care processes in detail to identify promising strategies that could be applied elsewhere, as an intervention with healthcare system A’s results would likely be cost-effective or even cost-saving based on typical annual costs ($100,000) and caseload (300 patients) for a falls care manager.

Our findings demonstrate that HTE can exist in the absence of a clearly demonstrable overall effect and suggest that HTE should be assessed in such circumstances [33]. Findings regarding HTE were robust to the analytic model used (Tweedie versus negative binomial model) but were not robust to the underlying data source for fall injury unit costs. Such differences point to the importance of a clear rationale for the selection of data sources for unit costs. In the current study, our primary data source for unit costs provided more detailed data on the time course of costs for a fall injury, with a large proportion of costs in hospitalized individuals occurring in the first three months after the injury. These data allowed for more accurate specification of costs over the short time horizon of this study.

In addition to observing HTE, we also observed substantial variability of costs across healthcare systems, a robust finding that persisted even with exclusion of healthcare system A. Adjusted total costs per PYF in control practices ranged from $1,529 to $3,684 for individual healthcare systems, which is more than twofold variation. Mediation analyses did not provide a clear explanation for observed differences; of the three candidate mediators, treatment intensity showed the most evidence of mediation.

Our findings have implications for the conduct of clinical trials, as well as for healthcare decisionmakers. A key consideration when assessing the generalizability of clinical trials has been understanding how study participants were selected and whether the clinical and demographic characteristics of these participants are representative of the target population for the intervention in question. Although trial reporting guidelines have also noted the importance of assessing generalizability of the healthcare systems in which participants are treated [34], less attention has been given to generalizability of healthcare systems included in multicenter clinical trials [35]. Decisions about which healthcare systems to include are particularly relevant for pragmatic trials with outcomes that include healthcare utilization. As the current analysis shows, such decisions will markedly affect both total costs and potential cost-effectiveness of an intervention. Such findings reinforce existing guidance to assess variation of costs when conducting economic evaluations within countries [1] and add to the more established literature looking at effects across countries [2–4].

This study carries with it certain limitations. First, we did not have access to actual cost data for participants, since the parent study did not collect these data. Consequently, we estimated costs based on participants’ reported healthcare utilization. This approach has the effect of focusing the analyses on differences in quantities of services used rather than on costs of those services. Second, healthcare costs that might have resulted from the STRIDE intervention, such as the costs of receiving physical therapy, were not measured, potentially biasing results in favor of the STRIDE intervention. Third, the current study focused on cost heterogeneity rather than cost-effectiveness analysis, which would determine if the STRIDE intervention represented “good value for money” as compared with other commonly accepted healthcare interventions. Although beyond the scope of this analysis, healthcare-system-specific cost-effectiveness analyses could be informative given the observed cost heterogeneity. Fourth, STRIDE interviews did not ask participants about pre-enrollment healthcare utilization for fall injuries, which would have allowed us to control for pre-existing cost trends at the various healthcare systems; although theoretically useful, from a practical standpoint, without prospective data collection (e.g., with falls calendars), such data are often limited by poor recall of participants for their prior events [36].

## Conclusions

We found that a small reduction in healthcare costs associated with the STRIDE intervention was driven by a single healthcare system. The finding of healthcare system cost heterogeneity was robust to inclusion or exclusion of the system in question. Even clinical trials limited to a single country should consider a formal assessment for healthcare system cost heterogeneity and HTE as part of their economic evaluation plan. This is particularly relevant for pragmatic trials which seek to enroll diverse populations and sites.

## Electronic supplementary material

Below is the link to the electronic supplementary material.

Supplementary Material 1

## Data Availability

The dataset analyzed in the current study is available in the National Institute on Aging repository (https://agingresearchbiobank.nia.nih.gov/ ) [20].

## Abbreviations

CHEERS: Consolidated Health Economic Evaluation Reporting Standards
FIMA: Fall injuries receiving medical attention
HTE: Heterogeneity of treatment effect
PYF: Person-year(s) of follow-up
STRIDE: Strategies to Reduce Injuries and Develop Confidence in Elders
US: United States
