# Supplementary material for: Costs of fall injuries in the STRIDE study: an economic evaluation of healthcare system heterogeneity and heterogeneity of treatment effect
Source: Cost Eff Resour Alloc. 2023 Aug 2;21:49. doi: 10.1186/s12962-023-00459-3 (PMC10399038; doi:10.1186/s12962-023-00459-3)
Supplement: Supplementary file 1 — Supplementary Material 1 [file 12962_2023_459_MOESM1_ESM.pdf]

Supplementary Appendix

Table of Contents

Supplementary Table 1. Counts and incidence rates of adjudicated serious fall injury events, hierarchically organized by most definitive injury type for each event.* 2

Supplementary Table 2. Unadjusted total costs per person-year of follow-up for FIMA and adjudicated serious fall injuries, by healthcare system and overall.......... 3

Supplementary Table 3. Adjusted total costs per person-year of follow-up for adjudicated serious fall injuries. 4

Supplementary Table 4. Omnibus (Type 3) Wald tests for cost of FIMA, comparing results using different data sources for costs.......... 5

Consolidated Health Economic Evaluation Reporting Standards (CHEERS) 2022 Checklist 6

Title.......... 6

Abstract.......... 6

Introduction.......... 6

Methods 6

Results 8

Discussion 8

Other Relevant Information 8

Acknowledgments 10

Supplementary Table 1. Counts and incidence rates of adjudicated serious fall injury events, hierarchically organized by most definitive injury type for each event.*

	Hospitalized Count (Incidence Rate)		Not hospitalized Count (Incidence Rate)	
**Injury type**	Intervention	Control	Intervention	Control
Hip fracture	39 (0.62)	43 (0.71)	1 (0.02)	0 (0.00)
Other fracture	53 (0.84)	57 (0.94)	109 (1.72)	118 (1.95)
Dislocation	0 (0.00)	0 (0.00)	5 (0.08)	3 (0.05)
Cut with evidence of closure	12 (0.19)	11 (0.18)	72 (1.14)	51 (0.84)
All other injuries	39 (0.62)	53 (0.88)	0 (0.00)**	0 (0.00)**

*All injury events are placed into the most definitive category for which they are eligible, ordered from most to least definitive: 1) hip fracture, 2) other fracture, 3) dislocation, 4) cut with evidence of closure, or 5) all other injuries. Incidence rates are per 100 person-years of follow-up (PYF). The intervention group had a total of 6338.31 PYF; the control group had a total of 6042.51 PYF.

**By definition, there are zero events in these cells, as events in the "all other injuries" category could only be considered a serious fall injury per STRIDE's definition if an overnight hospitalization was present.

Supplementary Table 2. Unadjusted total costs per person-year of follow-up for FIMA and adjudicated serious fall injuries, by healthcare system and overall.

		Unadjusted total costs/PYF			
	Total PYF	FIMA		Adjudicated SFI	
Healthcare system*		Intervention	Control	Intervention	Control
A	843	$1,615	$3,746	$956	$2,380
B	1,430	$2,472	$2,991	$1,088	$1,500
C	1,491	$2,321	$2,772	$1,147	$1,195
D	1,175	$1,886	$2,641	$895	$1,723
E	1,319	$1,875	$2,087	$866	$929
F	1,094	$1,957	$1,830	$1,156	$894
G	1,686	$1,765	$1,807	$802	$995
H	1,345	$2,574	$1,784	$1,340	$782
I	1,017	$2,006	$1,576	$1,000	$794
J	981	$1,793	$1,533	$972	$628
Overall	***12,381***	***$2,034***	***$2,289***	***$1,013***	***$1,173***

*Healthcare system letters are labeled “A” through “J” based on unadjusted total costs/PYF of their control practices for FIMA, ordered from highest to lowest.

Abbreviations: PYF, person-years of follow-up; FIMA, fall injuries with medical attention

Supplementary Table 3. Adjusted total costs per person-year of follow-up for adjudicated serious fall injuries.

	Total PYF	Adjusted total costs/PYF (95% CI)**	
**Healthcare system***		Intervention	Control
A	843	$1,013 ($565-$1,444)	$2,289 ($1,386-$2,833)
B	1,430	$1,111 ($767-$1,481)	$1,498 ($942-$2,054)
C	1,491	$1,155 ($1,030-$1,300)	$1,095 ($292-$1,899)
D	1,175	$871 ($317-$1,332)	$1,706 ($1,429-$1,956)
E	1,319	$864 ($573-$1,037)	$929 ($927-$930)
F	1,094	$1,249 ($810-$2,290)	$860 ($296-$1,341)
G	1,686	$728 ($293-$918)	$990 ($504-$1,566)
H	1,345	$1,333 ($994-$1,657)	$768 ($287-$1,301)
I	1,017	$995 ($481-$1,731)	$796 ($653-$933)
J	981	$1,047 ($431-$1,533)	$607 ($157-$1,279)
Overall	***12,381***	***$1,021 ($900-$1,134)***	***$1,145 ($965-$1,351)***

*Healthcare system letters are labeled "A" through "J" based on unadjusted total costs/PYF of their control practices for FIMA, ordered from highest to lowest.

**95% CI based on bootstrapped estimates from Tweedie model including covariates used in constrained randomization, treatment arm, and dummy indicators for healthcare systems and their interactions with treatment arm.

PYF, person-year of follow-up; CI, confidence interval; FIMA, fall injuries with medical attention

Supplementary Table 4. Omnibus (Type 3) Wald tests for cost of FIMA, comparing results using different data sources for costs.

**All healthcare systems**	**Bohl et al.**	**Hoffman et al.**
Effect	*p value*	*p value*
Treatment arm (intervention vs. control)	0.340	0.155
Healthcare system (dummy-coded)	0.035	0.037
Healthcare system by treatment arm interaction	0.090	0.258
Tertile of practice size (dummy-coded)	0.043	0.555
Study participants in practice were majority white race (vs. not)	0.429	0.464
Urban practice (vs. rural)	0.337	0.348

This table shows results for the primary analysis using cost data from Bohl et al.(22) , and a sensitivity analysis substituting data from Hoffman et al. (24).

Abbreviations: FIMA, fall injuries with medical attention

Consolidated Health Economic Evaluation Reporting Standards (CHEERS) 2022 Checklist

Title
1. Title: Identify the study as an economic evaluation and specify the interventions being compared.
   - Reporting location: Title page

Abstract
2. Abstract: Provide a structured summary that highlights context, key methods, results, and alternative analyses.
   - Reporting location: Abstract page

Introduction
3. Introduction (Background and Objectives): Give the context for the study, the study question, and its practical relevance for decision making in policy or practice.
   - Reporting location: Introduction section, all paragraphs

Methods
4. Health economic analysis plan: Indicate whether a health economic analysis plan was developed and where available.
   - Reporting location: Methods section, overview sub-section

5. Study population: Describe characteristics of the study population (such as age range, demographics, socioeconomic, or clinical characteristics).
   - Reporting location: Table 1

6. Setting and location: Provide relevant contextual information that may influence findings.
   - Reporting location: Introduction section, second paragraph; Methods section, overview sub-section

7. Comparators: Describe the interventions or strategies being compared and why chosen.
   - Reporting location: Introduction section, second paragraph.

8. Perspective: State the perspective(s) adopted by the study and why chosen.
   - Reporting location: Methods section, calculation of costs sub-section

9. Time horizon: State the time horizon for the study and why appropriate.
   - Reporting location: Methods section, calculation of costs sub-section

10. Discount rate: Report the discount rate(s) and reason chosen.
    - Reporting location: Methods section, calculation of costs sub-section

11. Selection of outcomes: Describe what outcomes were used as the measure(s) of benefit(s) and harm(s).
    - Reporting location: Not applicable

12. Measurement of outcomes: Describe how outcomes used to capture benefit(s) and harm(s) were measured.
    - Reporting location: Not applicable.

13. Valuation of outcomes: Describe the population and methods used to measure and value outcomes.
    - Reporting location: Not applicable.

14. Measurement and valuation of resources and costs: Describe how costs were valued.
    - Reporting location: Methods section, including data sources sub-section, outcomes sub-section, and calculation of costs subsection

15. Currency, price date, and conversion: Report the dates of the estimated resource quantities and unit costs, plus the currency and year of conversion.
    - Reporting location: Methods section, including overview sub-section and calculation of costs sub-section

16. Rationale and description of model: If modeling is used, describe in detail and why used. Report if the model is publicly available and where it can be accessed.
    - Reporting location: Not applicable.

17. Analytics and assumptions: Describe any methods for analyzing or statistically transforming data, any extrapolation methods, and approaches for validating any model used.
    - Reporting location: Methods section, statistical analysis sub-section

18. Characterizing heterogeneity: Describe any methods used for estimating how the results of the study vary for subgroups.
    - Reporting location: Methods section, statistical analysis sub-section

19. Characterizing distributional effects: Describe how impacts are distributed across different individuals or adjustments made to reflect priority populations.
    - Reporting location: Not reported.

20. Characterizing uncertainty: Describe methods to characterize any sources of uncertainty in the analysis.

- Reporting location: Methods section, statistical analysis sub-section

21. Approach to engagement with patients and others affected by the study: Describe any approaches to engage patients or service recipients, the general public, communities, or stakeholders (e.g., clinicians or payers) in the design of the study.

- Reporting location: Not reported.

Results

22. Study parameters: Report all analytic inputs (e.g., values, ranges, references) including uncertainty or distributional assumptions.

- Reporting location: Methods section, calculation of costs sub-section; Table 2

23. Summary of main results: Report the mean values for the main categories of costs and outcomes of interest and summarize them in the most appropriate overall measure.

- Reporting location: Table 3

24. Effect of uncertainty: Describe how uncertainty about analytic judgments, inputs, or projections affects findings. Report the effect of choice of discount rate and time horizon, if applicable.

- Reporting location: Table 3; Figure 1

25. Effect of engagement with patients and others affected by the study: Report on any difference patient/service recipient, general public, community, or stakeholder involvement made to the approach or findings of the study.

- Reporting location: Not reported.

Discussion

26. Study findings, limitations, generalizability, and current knowledge: Report key findings, limitations, ethical, or equity considerations not captured and how these could impact patients, policy, or practice.

- Reporting location: Discussion section, all paragraphs.

Other Relevant Information

27. Source of funding: Describe how the study was funded and any role of the funder in the identification, design, conduct, and reporting of the analysis.

- Reporting location: Title page

28. Conflicts of interest: Report authors' conflicts of interest according to journal or International Committee of Medical Journal Editors requirements.
    - Reporting location: Title page

Acknowledgments

The Data and Safety Monitoring Board

A 9-member Data and Safety Monitoring Board, appointed by the National institute on Aging, reviewed study's progress and safety data every 6 months, and included:

David M. Buchner MD MPH (Chair); Terry Fulmer PhD RN FAAN; Susan S. Ellenberg PhD; Bonita Lynn Beattie MPT MHA; Abby C. King PhD; Cynthia J. Brown MD MSPH FACP; Laurence Rubenstein MD MPH; MaryAnne Sterling CEA; Thomas R. Prohaska PhD; Lawrence Friedman MD

The STRIDE Study Team

Joint Principal Investigators:

Shalender Bhasin MB, BS, Communicating PI and Chair of the Steering and Publications Committees, and Central Project Management (Brigham and Women's Hospital, Harvard Medical School, Boston, MA)

Thomas M. Gill MD, PI and Chair of Protocol and Screening, Recruitment and Retention Committees (Yale School of Medicine, New Haven, CT)

David B. Reuben MD, PI and Chair of Intervention Committee (David Geffen School of Medicine at UCLA, Los Angeles, CA)

Central Project Management, Brigham and Women's Hospital, Boston, MA:

Shalender Bhasin MB BS (PI), Nancy K. Latham PT PhD (Study Director), Shehzad Basaria MD (Chief Medical Safety Officer), Thomas W. Storer PhD, Brooke F. Brawley MPA (cIRB Liaison), Richard Eder BA, Amy Larson MHA (Administrative Director), Lori Goehring BA (Study Manager), Molly Lukas BS, Scott Margolis MBA, Martha B. Carnie AS, Priscilla Gazarian PhD RN [University of Massachusetts Boston, Boston, MA], Maureen Fagan DNP, FNP-BC, FAAN [University of Miami Health System, Miami, FL], Lisa M. Quintiliani PhD [Boston University School of Medicine, Boston, MA]

Data Coordinating Center, Yale University, New Haven, CT:

DCC Principal Investigator: Peter Peduzzi PhD

James Dziura PhD, Denise Esserman PhD, Erich J. Greene PhD, Can Meng MS MPH, Heather Allore PhD, Charles Lu MS, Haseena Rajeevan PhD, Liliya Katsovich MA MBA CCRP, Rixin Wang PhD, Brian Funaro, Nancy Lorenze DNSc, Margaret Doyle MPH, Bridget Mignosa, Geraldine Hawthorne-Jones BS, Sui Tsang BS, Michael E. Miller PhD (Wake Forest), Thomas G. Travison PhD (Harvard), Peter Charpentier MPH (Chair of Data Management Committee), Katy Araujo MPH (Co-Chair of Data Management Committee)

Yale School of Medicine, New Haven, CT:

Geriatric Medicine:

Dorothy Baker PhD

Recruitment and Assessment Center:

Joanne M. McGloin MDiv, MS, MBA, Amy Shelton MPH, Eleni A. Skokos BS MS, Mara Abella, Luann M. Bianco BA, Rina Castro, Sabina Rubeck MPH, Karen Wu, David Nock BS, Crysta Collins AS BS, Kenneth Rando LCSW, Eloisa Martinez BS CCRP (UTMB)

School of Nursing, University of Minnesota

Siobhan McMahon PhD MPH GNP-BC (Nursing Director)

Clinical Trial Sites

Essentia Health, Duluth, MN Site

Principal Investigator: Stephen C. Waring, DVM, PhD, Fall Care Managers: Erica Chopskie RN, MPH and Heather Larsen RN, BA, Allise Taran MPH, Joseph Bianco MD, FAAFP, Margaret Hoberg CNP, Hillary Henzler-Buckingham BS, Austin Land

HealthCare Partners, El Segundo, CA

Site Principal Investigator: Jeremy Rich, DPM, Fall Care Manager: Vivian Chavez RN BSN, Christine Moore RN MA, Janelle Howe, Rosario Garcia, Samuel Ho MD, Yan Chen, MD

Johns Hopkins Medicine, Baltimore MD

Site Principal Investigator: Albert W. Wu MD MPH, Jeremy D. Walston, MD, Yuri Agrawal MD, Patti Ephraim MPH; Fall Care Manager: Tiffany Campbell BSN, RN, BMTCN, OCN; Johns Hopkins Community Physicians: Steven J. Kravet, MD MBA, Michael Albert MD, Bimal Ashar MD MBA, Bernhard Birnbaum MD, Sajida Chaudry MD, LaToya Edwards MD, Scott Feeser MD, Naaz A. Hussain MD, Amrish Joseph MD, Alice Lee MD, and Tara Scheck MD.

MercyOne Population Health Services Organization (Des Moines, IA*) and University of Iowa* (Iowa City, IA)

Site Principal Investigator: Robert B. Wallace MD, MSc, Co-Site Principal Investigator: Carri Casteel PhD, MPH; Fall Care Manager: Angela Shanahan BSN, RN-BC, Julie Weldon MSN RN, Anita Leveke BSN, RN CEN, Charles F. Keller MD, Jeffrey C. Reist BS, PharmD, BCPS; David Swieskowski MD

Michigan Medicine, University of Michigan, Ann Arbor, MI

Site Principal Investigator: Neil Alexander MD, Jocelyn Wiggins BM BCh, Fall Care Managers: Karen Burek RN MS ANP-BC and Tina Ledesma RN BSN, Linda V. Nyquist PhD, Nancy (Amby) Gallagher APRN-BC, PhD, Catherine Hanson BA

Mount Sinai Health System, New York, NY

Site Principal Investigator: Fred Ko MD MS, Albert L. Siu MD MSPH, Rosanne M. Leipzig MD PhD, Christian Espino BA, Ravishankar Ramaswamy MD MS, Fall Care Managers: Deborah West RN BS BA and Deborah Matza RN MPH C-IAYT

Partners Healthcare, Boston, MA

Site Principal Investigator: Patricia C. Dykes, RN, PhD, MA, Hilary Stenvig BS, Kety FlorGomes BA, BSN, RN, Taylor Christiansen BS, Alejandra Salazar PharmD RPh, Laura Frain MD MPH, Ariela Orkaby MD MPH, Jonathan Bean MD MPH, Fall Care Managers: Yvette Wells RN, BSN and Cathy Foskett BS, RN

Reliant Medical Group, Worcester, MA

Site Principal Investigator: Jerry H. Gurwitz MD; Falls Care Managers: Peggy Preusse RN and Anne McDonald RN; and Lawrence Garber MD, Allison Richards BA, Azraa Amroze BS, and Mary Ellen Stansky

University of Pittsburgh Medical Center, Pittsburgh, PA

Site Principal Investigator: Susan L. Greenspan, MD, Fall Care Manager: Mary Anne Ferchak RN BSN, Madeline Rigatti BS, Neil M. Resnick MD

University of Texas Medical Branch at Galveston, Galveston, TX

Site Principal Investigator: Elena Volpi MD PhD, Fall Care Manager: Summer R. Chapman MSN, RN, CCRP, Roxana Hirst MS CCRP, Eloisa Martinez BS CCRP, Mukaila Raji MD, MSc (Pharm), FACP, UTMB Clinical Practice Physicians: Megan A. Berman MD, Michael P. Binder MD, Cindy Chan MD, Matthew J. Hay, MD, Elizabeth A. Jaramillo MD MS, Michael T. Nguyen MD, Angela J. Shepherd MD, Lindsay K. Sonstein MD, UT Health Practicing Physicians: Julie S. Bortolotti MD, Amber M. Zulfiqar MD

STRIDE Committee Chairs:

Steering: Shalender Bhasin MB, BS

Protocol: Thomas M. Gill MD

Clinical Trial Sites: Jerry H. Gurwitz MD

Outcomes*:* Jay Magaziner PhD MSHyg (University of Maryland) and Albert L. Siu MD MSPH

Adjudication*:* David A. Ganz, MD PhD (UCLA and VA Greater Los Angeles Healthcare System)

Screening, Recruitment and Retention: Joanne M. McGloin M Div, MS, MBA and Thomas M. Gill, MD

Biostatistics: Peter Peduzzi PhD

Data Management and IT: Peter Charpentier MPH, Katy Araujo MPH

Intervention: David B. Reuben MD

Safety: Shehzad Basaria MD

Falls Care Managers: Siobhan McMahon PhD MPH GNP-BC (University of Minnesota);

National Patient and Stakeholder: Maureen Fagan DNP, FNP-BC, FAAN (Chair), Martha B. Carnie AS (Co-Chair), Catherine Hanson BA

Physical Components: Pamela W. Duncan PhD, PT (Wake Forest University) and Thomas W. Storer PhD

FCM Training: Chad Boult MD MPH MBA (Johns Hopkins) and Priscilla Gazarian PhD RN

Ancillary Studies: James S. Goodwin MD (UTMB) and Todd M. Manini PhD (University of Florida)

Publications: Shalender Bhasin MB, BS and Peter Peduzzi PhD

Wake Forest University, Winston-Salem NC

Kevin P. High MD MS, Lea N. Harvin BS, Cynthia L. Stowe MPM

National Institute on Aging, Bethesda, Maryland:

Program Officer: Sergei Romashkan MD, Scientific Officer: Rosaly Correa-de-Araujo MD MS PhD; Other Program Staff: Lyndon Joseph PhD, Marcel E. Salive MD MPH, Evan C. Hadley MD

Patient Centered Outcomes Research Institute (PCORI), Washington, D.C.

Steven B. Clauser PhD MPA

Funding Support

The STRIDE study was funded primarily by the Patient Centered Outcomes Research Institute (PCORI), with additional support from the National Institute on Aging (NIA) at NIH. Funding is provided and the award managed through a cooperative agreement (5U01AG048270) between the NIA and the Brigham and Women's Hospital. The project is part of the Partnership for Fall Injuries Prevention between the NIA and PCORI. This research is partially supported by the Boston Claude D. Pepper Older Americans Independence Center at Brigham and Women's Hospital (P30AG013679) and Harvard Catalyst | The Harvard Clinical and Translational Science Center (National Center for Research Resources and the National Center for Advancing Translational Sciences, National Institutes of Health Award UL1TR001102) and financial contributions from Harvard University and its affiliated academic healthcare centers. Support was also provided by the Claude D. Pepper Older Americans Independence Centers at UCLA (P30AG028748); Yale (P30AG021342); Mt Sinai (P30AG2874106); UTMB (P30AG024832), University of Michigan (P30AG024824) and Wake Forest (P30AG021332). Mt Sinai also received support through a grant from the New York Academy of Medicine. Additional support at Yale University was provided by the NIH/National Center for Advancing Translational Sciences Clinical and Translational Science Awards program (UL1TR000142) and an Academic Leadership Award (K07AG043587) to Dr Gill from the National Institute on Aging. Dr. McMahon was supported by grants KL2TR000113 and UL1TR000114. The University of Michigan also received support from Michigan Medicine, its academic healthcare system. The content of this publication is solely the responsibility of the authors and does not necessarily represent the official views of the National Institutes of Health.
